# Wheat TaMs1 is a glycosylphosphatidylinositol-anchored lipid transfer protein necessary for pollen development

**DOI:** 10.1186/s12870-018-1557-1

**Published:** 2018-12-05

**Authors:** Allan Kouidri, Ute Baumann, Takashi Okada, Mathieu Baes, Elise J. Tucker, Ryan Whitford

**Affiliations:** 1 University of Adelaide, School of Agriculture, Food and Wine, Waite Campus, Urrbrae, South Australia 5064 Australia; 2 Commonwealth Scientific and Industrial Research Organization, Agriculture and Food, Waite Campus, Urrbrae, South Australia 5064 Australia

**Keywords:** Wheat, LTP, Glycosylphosphatidylinositol-anchored lipid transfer protein, Sporopollenin, Pollen exine, Male sterility

## Abstract

**Background:**

In flowering plants, lipid biosynthesis and transport within anthers is essential for male reproductive success. *TaMs1,* a dominant wheat fertility gene located on chromosome 4BS, has been previously fine mapped and identified to encode a glycosylphosphatidylinositol (GPI)-anchored non-specific lipid transfer protein (nsLTP). Although this gene is critical for pollen exine development, details of its function remains poorly understood.

**Results:**

In this study, we report that *TaMs1* is only expressed from the B sub-genome, with highest transcript abundance detected in anthers containing microspores undergoing pre-meiosis through to meiosis. *β-glucuronidase* transcriptional fusions further revealed that *TaMs1* is expressed throughout all anther cell-types. *TaMs1* was identified to be expressed at an earlier stage of anther development relative to genes reported to be necessary for sporopollenin precursor biosynthesis. In anthers missing a functional *TaMs1* (*ms1c* deletion mutant), these same genes were not observed to be mis-regulated, indicating an independent function for TaMs1 in pollen development. Exogenous hormone treatments on GUS reporter lines suggest that *TaMs1* expression is increased by both indole-3-acetic acid (IAA) and abscisic acid (ABA). Translational fusion constructs showed that TaMs1 is targeted to the plasma membrane.

**Conclusions:**

In summary, *TaMs1* is a wheat fertility gene, expressed early in anther development and encodes a GPI-LTP targeted to the plasma membrane. The work presented provides a new insight into the process of wheat pollen development.

**Electronic supplementary material:**

The online version of this article (10.1186/s12870-018-1557-1) contains supplementary material, which is available to authorized users.

## Background

Wheat (*Triticum aestivum* L.) is one of the most staple food crops and accounts for 20% of human daily protein and food calories (FAOSTAT, 2017). The demand for wheat is predicted to increase 60% by 2050 compared with 2010. Thus, an increase of the global yield gain from the current rate of 1% (2001–2010) to 1.6% per year (2010–2050) is required. Male reproductive development is a key factor for grain yield. Pollen grains are encapsulated by a complex multiple-layered cell wall termed exine, which forms a physical barrier against a variety of biotic and abiotic stresses [1]. Pollen exine mainly consists of sporopollenin, a highly resistant biopolymer providing a rigid exoskeleton, which in grass species is additionally covered by tryphine, a mixture of phenolic, protein and fatty acid derivatives [2, 3].

The highly recalcitrant nature of sporopollenin to chemical degradation has proven a great challenge in unravelling its biochemical composition. However, the underlying genetics of pollen wall development has been intensively investigated through the use of exine-defective mutants in model plants such as *A. thaliana *and rice among other species [1]. These genetic analyses indicate that sporopollenin biosynthesis consists of three conserved metabolic pathways and transport processes. The first of these involves production of waxes and various lipid-based compounds from precursors including phospholipids, fatty acids and alcohols. This pathway includes fatty acid hydroxylases such CYP703A3 [4, 5] and CYP704B2 [6] from the conserved P450 gene family. Additionally, MALE STERILITY 2 (MS2) from *A. thaliana* [7] and its rice orthologue DEFECTIVE IN POLLEN WALL (DPW) [8] encode fatty acid reductases which have been shown to be essential for pollen exine formation.

The second conserved pathway involves phenolic compound biosynthesis, an important component of exine and tryphine [9]. Phenolics are synthesized from fatty acid substrates by fatty-acyl-CoA synthetases (ACOS5) [7], polyketide synthetases (OsPKS1) and tetraketide α-pyrone reductases (TKPR) [10].

The third conserved pathway involves polysaccharide metabolism whereby the timing of callose biosynthesis and degradation facilitates pollen coat formation [11, 12].

Newly synthesized sporopollenin precursors are then translocated from the tapetal cell layer to developing microspores. How sporopollenin precursors are allocated for pollen coat formation remains unclear. Studies reveal that ABCG15, encoding an ATP-binding cassette (ABC) transport protein, in addition to non-specific lipid transfer proteins, play roles in sporopollenin precursor transport [13, 14]. Additionally, it was shown that *A. thaliana* type III-LTPs allocate and incorporate lipidic compounds to the pollen wall [15]. More recently, a wheat gene termed *TaMs1* encoding a glycosylphophatidylinositol (GPI) Lipid Transfer Protein was demonstrated to be required for wheat male fertility [16, 17].

Members of the non-specific lipid transfer protein (nsLTP) gene family have been identified in most plant species. They exhibit a range of expression patterns across different developmental stages. This is reflected by their potential involvement in numerous biological processes, including cutin biosynthesis [18], pathogen defense response [19], long distance signaling [20, 21], seed maturation [22], and pollen tube adhesion [23].

nsLTPs have the ability to shuttle lipids between membranes in vitro [24]. They are part of a plant specific prolamin superfamily, identifiable by an eight conserved cysteine motif (8CM) backbone, low molecular mass and 4 to 5 alpha-helices [25, 26]. The conserved cysteine domain has the following pattern: C-Xn-C-Xn-CC-Xn-CXC-Xn-C-Xn-C, with cysteine residues required for the formation of four disulphide bridges [27]. In this context the disulfide bridges stabilize a hydrophobic cavity with the ability to bind various lipids and other hydrophobic compounds in vitro [28]. Most nsLTPs also possess an N-terminal signal peptide targeting the proteins to the apoplastic space via the vesicular secretory pathway. nsLTPs can also contain a conserved C-terminal motif subject to post-translational modification. This motif is recognised by glycophosphatidylinositol (GPI) transamidases in the lumen of the endoplasmic reticulum (ER) whereby it is cleaved and replaced by a GPI moiety. This GPI moiety anchors the protein to the extracellular side of the plasma membrane. GPI-anchored nsLTPs can be released from the membrane by specific phospholipases that cleave the GPI molecule [29].

Genome wide analysis of nsLTPs in rice and *Arabidopsis* reported 77 and 79 nsLTPs, respectively [30]. In wheat 156 putative nsLTPs were retrieved by EST data mining [31]. nsLTPs are categorized into at least nine types, distinguished based on intron position, inter-cysteine spacing and the presence of a GPI-anchor motif [31, 32]. Among the nine reported types, GPI-anchored nsLTPs, type G, are the most represented in rice and *A. thaliana* [30].

In this study, we investigated the biological function of TaMs1 during pollen exine formation. We report evidence for spatio-temporally restricted expression of *TaMs1* in anthers undergoing microsporogenesis. TaMs1 is shown to be expressed earlier than many genes required for sporopollenin-biosynthesis. Finally, we demonstrate the importance of both signal peptide and pro-peptide GPI anchor for TaMs1 subcellular localization as indicative of a role in lipidic transport. Our results provide new insights into mechanisms of pollen development.

## Methods

### Plant materials and growth conditions

Wheat cultivars Chris and Chris-EMS mutagenized lines FS2 (*ms1d*) were used for cytological examination and expression profiling [33]. Plants were sown at 5 to 6 plants per 6 L (8 in. diameter) pot containing soil mix. The soil mix consisted of 75% (v/v) Coco Peat, 25% (v/v) nursery cutting sand (sharp), 750 mg/L CaSO_4_.2H_2_O (gypsum) 750 mg/L Ca(H_2_PO_4_)_2_.H_2_0 (superphosphate), 1.9 g/L FeSO_4_, 125 mg/L FeEDTA, 1.9 g/L Ca(NO_3_), 2.750 mg/L Scotts Micromax micronutrients, and 2.5 g/L Osmocote Plus slow release fertilizer (16:3:9) (Scotts Australia Pty. Ltd.). pH was adjusted to between 6.0 and 6.5 using 2 parts agricultural lime to 1 part hydrated lime. Potted plants were grown either in controlled environment growth rooms at 23 °C (day) and 16 °C (night) or similarly temperature moderated glasshouses in which photoperiod was extended using 400 W high pressure sodium lamps in combination with metal halide lamps to 12 h over winter months.

### Expression analysis by qRT-PCR

Total RNA was isolated using ISOLATE II RNA Mini Kit (Bioline, Sydney, Australia) from wheat tissues: roots, shoot and glume, lemma, palea, pistil, and anthers containing microspores from pre-meiosis to maturity. Quantitative real-time PCR was perform according to Burton et al.*,* (2004) [34] using the primer combinations shown in Additional file 1. Anthers containing developing microspores were staged by acetocarmine staining. 0.6 μg of RNA was used to synthesise oligo(dT)-primed first strand cDNA using the superscript IV reverse transcriptase (Thermo Fisher Scientific, Melbourne, Australia). 2 μL of the RT product diluted 1:20 was then used as template for conventional and quantitative real-time PCR. *TaGAPdH, TaActin and Ta14–3-3* were used as reference genes.

### Histochemical GUS staining and cytological examination

The construct pTOOL36-*TaMs1::gusplus* [16] was transformed into wheat (cv. Fielder) using *Agrobacterium tumefaciens* according to Ainur et al.*,* 2014 [35]. GUS activity in transgenic lines from leaves, roots and anthers containing microspores at pre-meiosis to maturity were analysed by histochemical staining using 5-Bromo-4-chloro-3-indolyl-beta-D-glucuronic acid (Gold Biotechnology, Inc). Samples were incubated in a 1 mM X-Gluc solution in 100 mM sodium phosphate, pH 7.0, 10 mM sodium ethylenediaminetetraacetate, 2 mM FeK_3_(CN)_6_, 2 mM K_4_Fe(CN)_6_ and 0.1% Triton X-100. After vacuum infiltration at 2600 Pa for 20 min, samples were incubated 72 h at 37 °C.

Samples were incubated in fixative solution of 4% sucrose, 1x PBS, 4% paraformaldehyde, 0.25% glutaraldehyde, at 4 °C overnight. Samples were subsequently dehydrated in an ethanol series of increasing concentration (30, 50, 70, 85, 90, 95 and 100%). Tissues were then embedded in *Technovit®* resin, microtome sectioned at 8–14 μm, counter-stained with ruthenium red and then mounted in DPX solution (Sigma, St. Louis, MO). Sections were observed using standard light microscopy on a LEICA DM1000 microscope coupled with a CCD camera. The precipitated product from the β-glucuronidase reaction appears blue under bright field and pink under dark field.

### Promoter analysis

NewPLACE [36], an online database of plant *cis*-acting regulatory DNA elements (*cis-*element) was used to identify putative *cis*-elements in the promoter regions of *TaMs1* and its homeologues.

### Hormone response assays

Plants were treated with indole-3-acetic acid (IAA) (PytoTechnology Lab., Lenexa, USA) and abscisic acid (ABA) (Sigma-Aldrich, Sidney, Australia). Hormone stock solutions were made with 100% ethanol. Wheat tillers were collected and dipped in hormone solutions for 9 h containing either 100 μM IAA or 100 μM ABA, to a final concentration of 0.05% ethanol. A 0.05% ethanol solution was used as a control treatment. For the drought treatment, plants were well watered until the stage of flag leaf emergence and water withheld until wilting. After sample collection, plants were re-watered. The effects of the cyclic drought treatment was assessed from the percentage of fertility of three spikes from well-watered and treated plants calculated according to Tucker et al. (2017) [16].

GUS activity in anthers from treated transgenic lines was analyzed by histochemical staining using X-gluc as previously described. Anthers containing developing microspores were staged by acetocarmine staining. Six spikes were used for each treatment.

### Expression analysis from RNA-sequencing

Anther tissues of wheat Cornerstone fertile (WT) and sterile (*ms1c*), 4 replicates each, were isolated from anther containing pre-meiotic microspores to binucleate microspores. Tissue samples were frozen in liquid nitrogen immediately post collection. Total RNAs were extracted using RNeasy Plant mini kit (Qiagen). Each sample was used to create libraries that were deep-sequenced using the Illumina**™** Hi-Seq 2500 system to generate 100 bp, paired-end reads. Reads were trimmed based on quality scores (Phred score ≥ 15) and adapter sequences were removed. Reads were mapped to the IWGSC RefSeqv0.4 wheat genome assembly [37] using TopHat2.0 with default parameters except for maximum intron size: 50,000 bp; minimum intron size: 20 bp; 1 mismatch/100 bp allowed. Aligned reads were assembled with CuffLinks [38] and then quantified and normalized with Cuffnorm. Normalized expression is expressed in FPKM, read per kilo base per million reads. Significance of differences in gene expression between WT and *ms1c* for the genes of interest in this study were calculated using Student’s t-test two-tailed.

### Amino-acid sequence analysis

TaMs1 amino acid sequence were tested for the presence of a putative signal peptide using SignalP 4.1 [39]. Additionally, the presence of a GPI-anchor domain was predicted using big-PI plant predictor [40], PredGPI [41] and GPI-SOM [42].

### Subcellular localization of TaMs1

The fusion construct mCherry-TaMs1 was synthetized by GeneScript® and inserted between the BamHI and KpnI sites of pUC57-Kan to generate pUC57-mCh-TaMs1. *TaMs1* coding sequence from wheat cv. Chris was used as template and the mCherry reporter was inserted between Q24 and P25 of the TaMs1 protein. pUC57-mCh-TaMs1 was digested by BamHI/KpnI and the fragment containing mCh-TaMs1 was inverted and inserted between the maize ubiquitin promoter (ZmUbi) and RuBisCo terminator resulting in p*ZmUbipro*::mCh-TaMs1. The constructed p*ZmUbipro*::mCh-TaMs1 was used for transient expression in epidermal onion cells as well as wheat protoplasts according to Bart et al., 2006 and Shan et al., 2014 [43, 44]. p*ZmUbipro*::mCh was used as a transformation control. Confocal images were captured with a Nikon A1R laser scanning microscope (Nikon Instruments Inc., U.S.) coupled to a DS-Ri1 CCD camera. A 488 nm laser was used for GFP fluorescence (excitation: 488.0 nm; emission: 525.0 nm) detection and the 561 nm laser for RFP fluorescence (excitation: 561.1 nm; emission: 595 nm) detection. Plasmolysis was performed using 0.8 M mannitol.

### Callose staining

Anthers samples were collected from male fertile plants (wild type) and sterile plants (*ms1d*) containing meiotic microspores (meiosis I, dyad and tetrad) and uninucleate microspores. Developmental stages were determined by acetocarmine staining or cytological examination. Callose wall staining was performed by squashing anthers in a drop of aniline blue solution (0.1% aniline blue in 0.1 M phosphate buffer pH 7.0) [45]. Both bright-field and fluorescence microscopy were performed using a Nikon ECLIPSE NiE optical microscope.

## Results

### *TaMs1* is an anther-specific gene expressed early during anther development

**Fig. 1:**
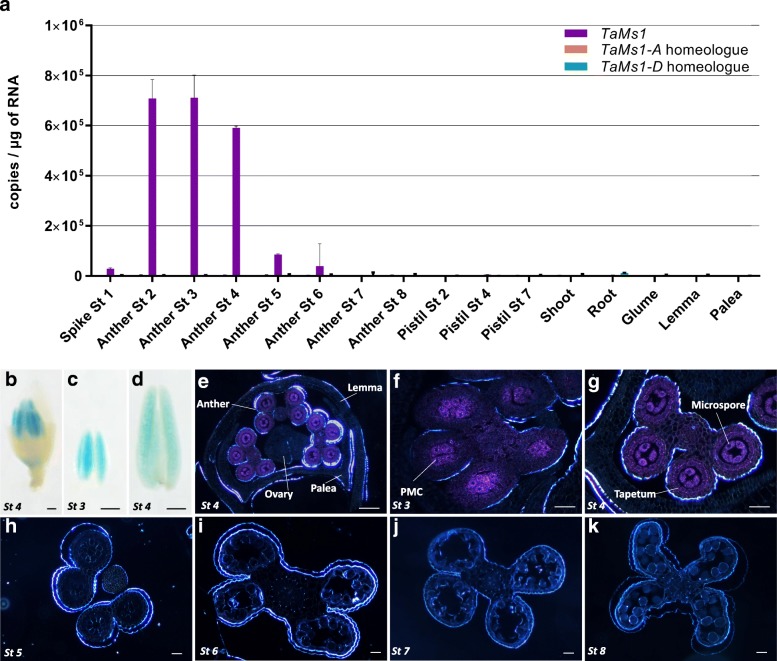
*TaMs1* expression is anther-specific and predominantly within pre-meiotic to meiotic microspores. qRT-PCR expression profiling of *TaMs1* and its homeologues (**a**) in anthers containing pre-meiotic microspores to mature pollen, pistil, shoots, roots, glume, lemma and palea. St1, Spike white anthers; St2, archesporial cells; St3, pre-meiotic pollen mother cells; St4, meiotic microspores; St5, early uninucleate; St6, late uninucleate; St7, binucleate; St8, mature pollen. Error bars reflect standard error of three independent tissue replicates (*n* = 3). GUS activity in whole mount tissue samples (**b**-**d**), transverse section of floret (**e**) and anthers (**f**-**k**) in transgenics expressing *TaMs1::gusplus*. Scale bars: **b**-**d**, 100 μm; **d**, 200 μm; **e**-**j**, 50 μm

*TaMs1* transcripts were not detected in pistils, shoots, roots, glume, lemma or palea, however, transcripts were enriched in anther tissues with their abundance peaking when microspores were at pre-meiosis to meiosis, stage (st) 2 to 4 (Fig. 1a). *TaMs1* expression decreased significantly in anthers containing uninucleate microspores (st 5 and 6). Additionally, only the B homeologue was detected, indicating only this sub-genome is transcribed.

Furthermore, analyses of *TaMs1* promoter activity in transgenic wheat cv. Fielder were performed using *TaMs1:*:*gusplus* transcriptional fusion constructs. Similar to the qRT-PCR results, GUS activity was observed exclusively in anthers containing microspores at pre-meiosis (st 3) till meiosis (st 4) (Fig. 1b-g). Transverse sections of anthers containing pre-meiotic microspores (st 3) revealed GUS activity predominantly in Pollen Mother Cells (PMCs) with weak detection in all other anther cell types (Fig. 1f). Whereas, in anthers containing early meiotic microspores (st 4), high GUS activity was detected both in microspores and tapetal cells, and to a lesser extent in other tissues of the anther (Fig. 1g). No GUS activity was detected in anther transverse sections from uninucleate microspores to pollen maturity (st 5 to 8) (Fig. 1h-k).

Because callose metabolism coincides with *TaMs1* expression profile, we tested whether TaMs1 is involved in callose formation during meiosis by aniline blue staining. Callose is deposited onto the meiocyte cell wall during meiosis [see Additional file 2] and then degraded at microspore tetrad (right panels in Additional file 2), subsequently releasing uninucleate microspores. No difference was observed in the pattern, quantity or timing of callose deposition between WT and *ms1d*, suggesting no functional involvement of TaMs1 in callose deposition during meiosis.

### Effect of exogenous hormones on *TaMs1* expression

**Fig. 2:**
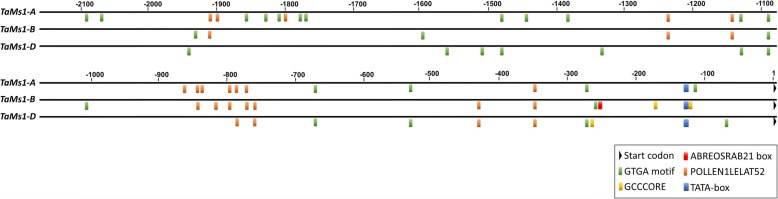
Distribution of some *cis*-acting elements in the promoter sequence of *TaMs1* and its homeologues. Selected pollen specific and hormone responsive *cis*-acting were retrieved in 2 kb *TaMs1* and its homeologues promoter sequences. The three homeologue promoter regions were annotated after sequence alignments. Start codons and putative GTGA motifs, ABREOSRAB21 and GCCCORE, TATA-boxes, POLLEN1LELAT52 *cis-*elements are represented by the different symbol as indicated. Numbering is from the first base of translations start site (+ 1)

To investigate the regulation of *TaMs1* present on chromosome 4B, we identified in silico putative *cis*-regulatory elements in the 2 kb the promoter region of *TaMs1* and its homeologues (Fig. 2). Two types of *cis*-elements related to pollen specific expression, GTGA motif and POLLEN1LELAT52 [46], were detected using the newPLACE tool [36]. All three homeologues contained putative GTGA motif and POLLEN1LELAT52 elements in their promoter regions. The GTGA motif was enriched in the *TaMs1-A* promoter region with 16 occurrences, while 11 and five occurrences were identified in *TaMs1-D and TaMs1-B,* respectively. *TaMs1-A and TaMs1-B* contained respectively 12 and ten copies of POLLEN1LELAT52 element, while only four copies were identified in *TaMs1-D* promoter region.

Two hormone responsive elements were identified in *TaMs1-B* promoter region, including two GCCCORE-boxes, a jasmonate/ethylene responsive element, located at − 103 bps and − 155 bps from the start codon, and ABREOSRAB21, an ABA responsive element activator of transcription [47], at − 234 bps. Interestingly, the ABREOSRAB21 was identified only on *TaMs1-B* promoter region. In addition, *TaMs1-D* promoter region contained only one putative GCCCORE element located at − 237 bps and none were identified in the *TaMs1-A* promoter region.

**Fig. 3:**
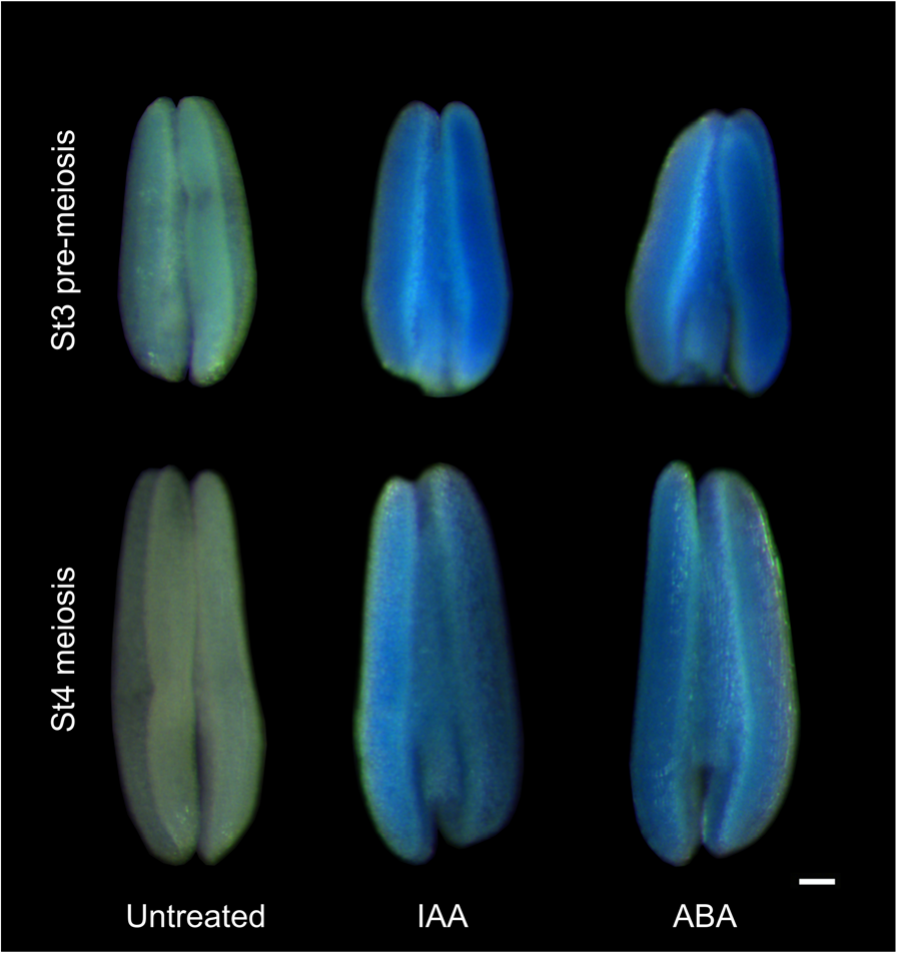
*TaMs1* promoter activity in response to exogenous IAA and ABA treatment. GUS activity in whole mount anther samples in transgenic expressing *TaMs1::gusplus* in response to hormonal treatment using IAA and ABA (9 h hormonal treatments). Anther samples were GUS-stained for 48 h at 37 °C. St3, pre-meiotic pollen mother cells; St4, meiotic microspores. Scale bars: 100 μm

Because the distribution of hormone response *cis*-elements in *TaMs1* promoter region differed relative to its homeologues, we first investigated the effects of exogenous hormones on *TaMs1* expression using *TaMs1::gusplus* lines. Differences in GUS activity between treatments was determined by altering staining time. Firstly, blue color saturation for the untreated *TaMs1::gusplus* line was found to occur at approximately 72 h, therefore GUS staining for treatments was stopped when differences between these and the control were first observed. This typically occurred at approximately 48 h of GUS staining. *TaMs1::gusplus* anthers containing pre-meiotic and meiotic microspores were more intensely stained after nine hours of indole-3-acetic acid (IAA) and abscisic acid (ABA) treatment relative to mock treated controls (Fig. 3), suggesting *TaMs1* is transcriptionally activated by these hormones. No differences in GUS activity were observed in response to jasmonic acid (JA) and gibberellic acid (GA_3_) treatments (data not shown).

### *TaLTPG1* is expressed earlier than other genes deemed necessary for pollen exine formation

**Fig. 4:**
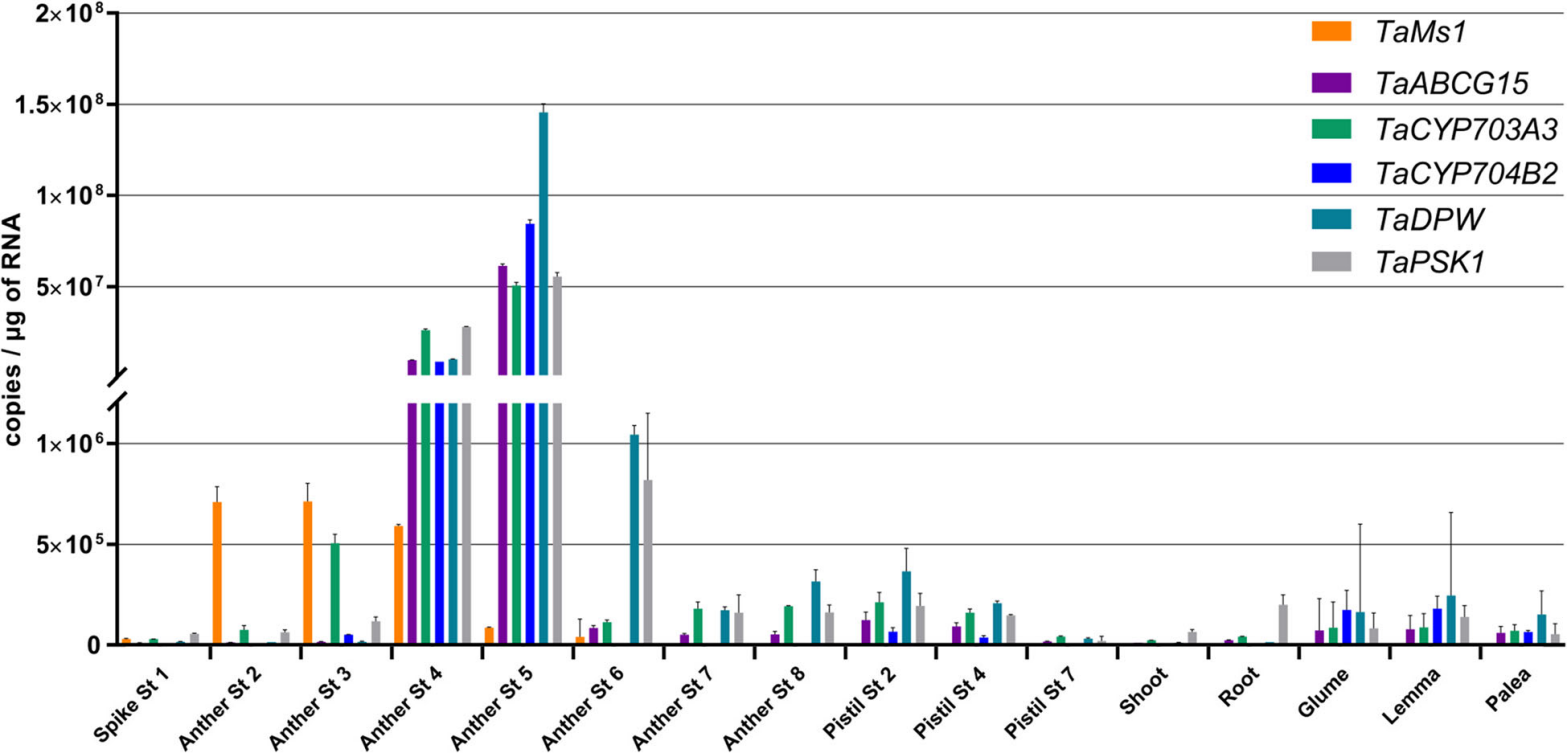
Sporopollenin biosynthetic genes *TaABCG15, TaCYP703A3*, *TaCYP704B2, TaDPW* and *TaPSK1*, are expressed in anthers after *TaMs1*. qRT-PCR expression profile of *TaABCG15, TaCYP703A3, TaCYP704B2, TaDPW and TaPSK1* in anthers containing pre-meiotic microspores to mature pollen, pistil, shoot, root, glume, lemma and palea. St1, Spike white anthers; St2, archesporial cells; St3, pre-meiotic pollen mother cells; St4, meiotic microspores; St5, early uninucleate; St6, late uninucleate; St7, binucleate; St8, mature pollen. Error bars reflect standard error of three independent tissue replicates (*n* = 3)

*TaMs1* is expressed within anthers containing sporogenous cells (stage 2), an early stage of anther development (Fig. 1f). To better understand *TaMs1*’s function, we investigated the timing of its expression relative to wheat orthologues of rice sporopollenin-biosynthetic genes such as *TaABCG15, TaCYP703A3*, *TaCYP704B2*, *TaDPW* and *TaPSK1* [4, 6, 48] (Fig. 4). Transcripts for each of these genes were preferentially detected in anther samples containing meiotic to uni-nucleate microspores (stage 4 and 5).

### *TaMs1* knock-out does not affect the expression level of genes involved in anthers and pollen wall development at meiosis stage

**Fig. 5:**
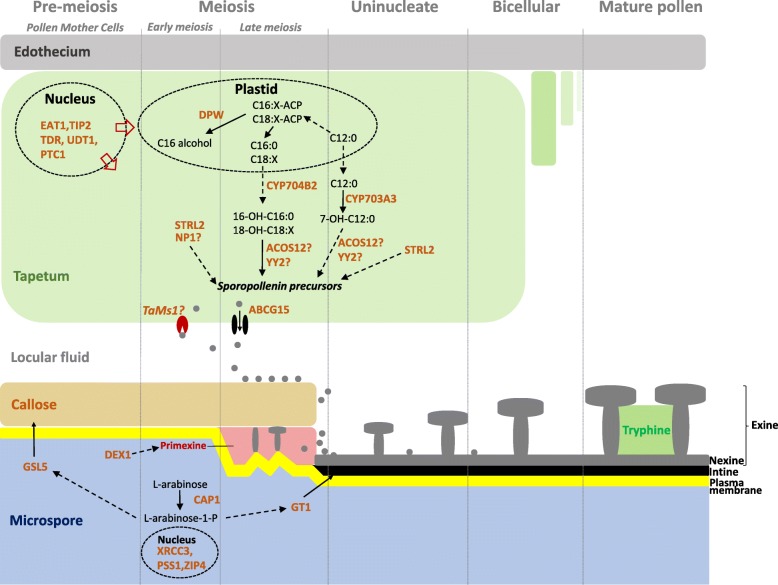
Current model of pollen development and metabolic network of exine formation in rice and *A. thaliana.* (Adapted from Ariizumi and Toriyama (2011) and Zhang et al. (2016) with modification (License number: 4286200743277 and 4286240859506)). For full names of the genes/enzymes refer to Additional file 3

**Fig. 6:**
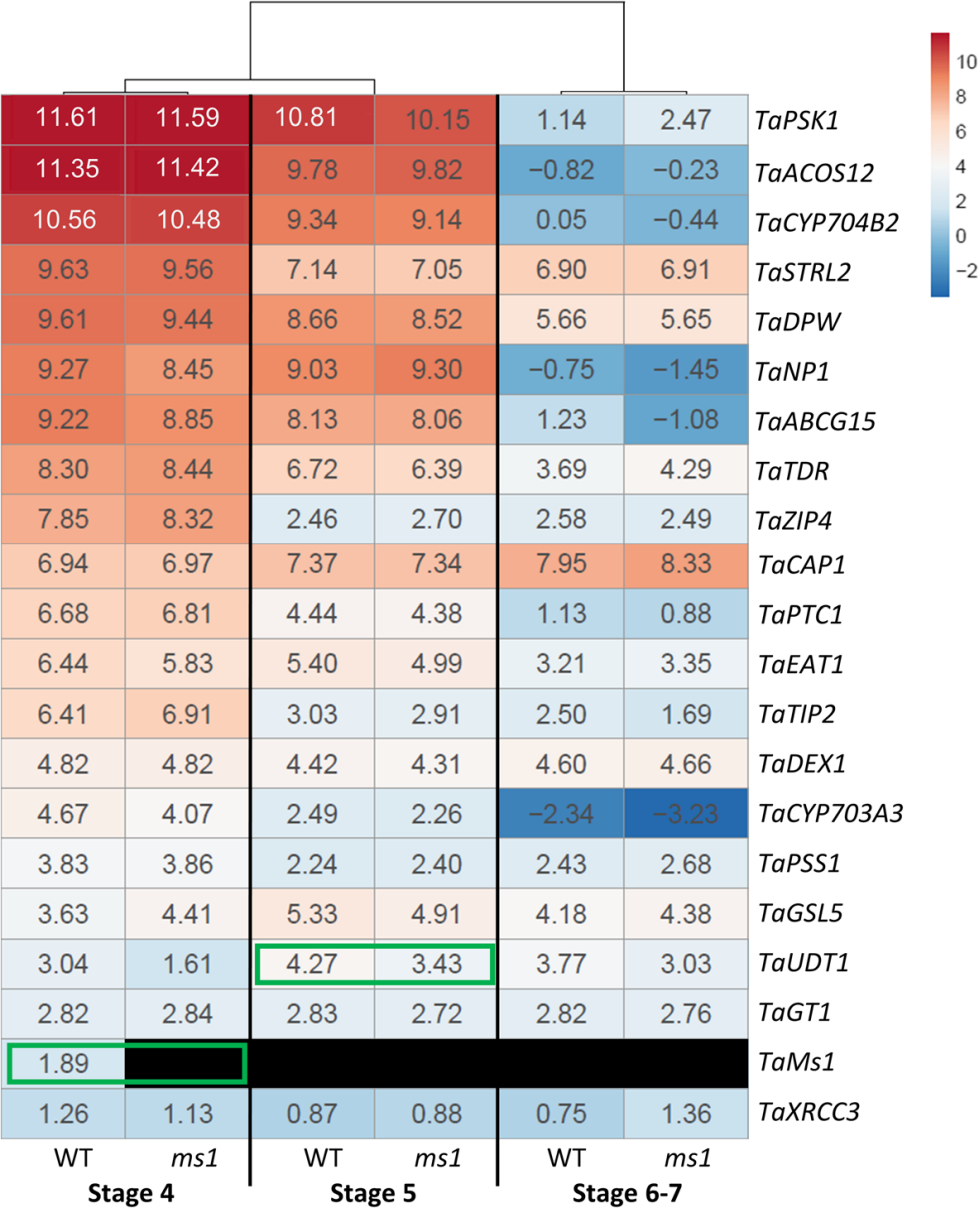
Expression analysis of genes related to anther and pollen wall synthesis between Wild-Type and *ms1*. Expression value indicates log2 FPKM from RNA-seq data. The color bar represents the relative signal intensity value, red indicates higher while blue represents lower expression and black indicates no expression detected. Stage 4, meiotic microspores; Stage 5, early uninucleate; Stage 6, late uninucleate. Stage 7, binucleate. Hierarchical clustering of samples was obtained using McQuitty correlation. Green squares denote the values significantly different between WT and *ms1* (*P* < 0.05) by student’s t-test analysis

Inter-dependent regulatory relationships of genes during male reproductive development have been reported in rice amongst other species. For example, rice mutants for genes deemed necessary for pollen formation typically show differential expression patterns for many genes identified to be involved in pollen exine formation [49]. We aimed to determine if this holds true in wheat, by examining gene expression profiles, in Wild Type (WT) versus *ms1* anthers, for 20 putative wheat orthologues to rice sporopollenin biosynthetic genes reported to be necessary for male fertility. Genes were identified firstly based on reports of male sterility mutations in rice, and then based on whether they had been functionally characterized and shown to be essential for anther development and pollen wall formation (Fig. 5; Additional file 3; Fig. 6).

Surprisingly, none of the selected genes displayed abnormal expression in *ms1* anthers containing meiotic microspores (stage 4), with the exception of *TaMs1* (Fig. 6). In *ms1* anthers containing uni-nucleate microspores (stage 5), only *UNDEVELOPED TAPETUM1* (*TaUDT1)* was significantly down-regulated relative to WT. However, its expression was not altered across other stages of pollen development. No significant difference in gene expression could be observed for the other sporopollenin biosynthetic genes at this stage. The rice *UDT1,* a bHLH transcription factor, has been reported to be critical for early tapetum development and PMC meiosis [50]. At stage 6 and 7, expression levels of sporopollenin biosynthetic genes were not affected by the *Tams1* mutation.

### TaMs1 protein is localized to the plasma membrane

**Fig. 7:**
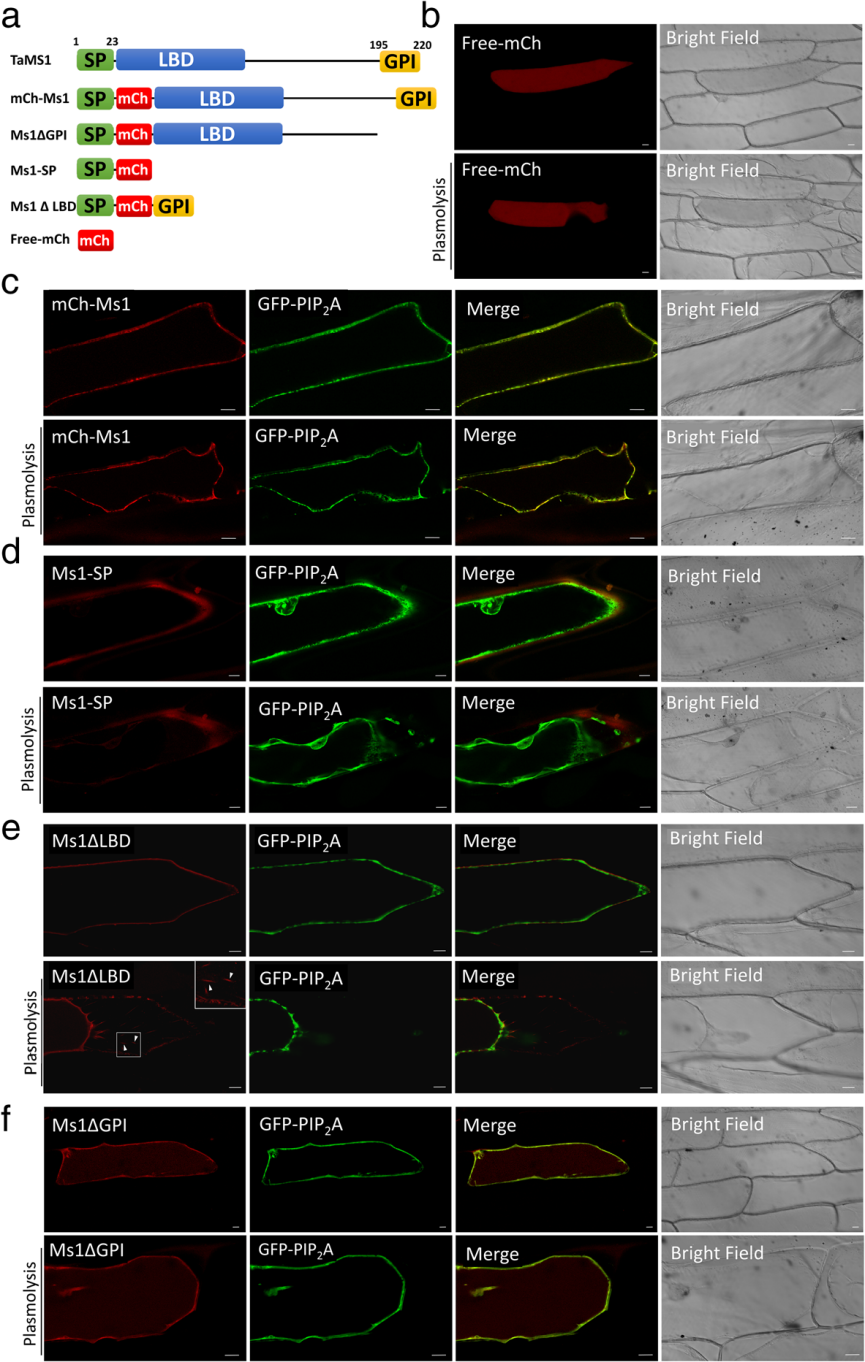
TaMs1 is targeted to the plasma membrane. **a** Schematic representation of the TaMs1 full length pre-protein and translational reporter fusion constructs used for epidermal onion cell transient expression assay (**b**) Cytosolic fluorescence of free mCh. **c**-**f** Co-expression of GFP- PIP_2_A plasma membrane marker and TaMs1 full length or truncated proteins with and without plasmolysis. Scale bars = 20 μm

Computational analysis of TaMs1 primary polypeptide predicts the presence of (i) an N-terminal signal secretory peptide (SP) 23 amino acids in length that is expected to target the mature protein to the secretory pathway, (ii) followed by an eight cysteine motif characteristic of LTPs’ lipid binding domain (LBD) consensus, (iii) and a C-terminal transmembrane domain that is predicted to be post-translationally cleaved and replaced with a GPI-anchor (Fig. 7a). The TaMs1 protein defined by its three putative motifs, SP-LBD-GPI, is predicted to be secreted via the vesicular pathway and tethered to the extracellular side of the plasma membrane by a GPI moiety. In order to confirm TaMs1’s sub-cellular localization in vivo, TaMs1 was fused with mCherry (mCh) and transiently expressed in onion epidermal cells.

Free mCh Fluorescence was observed to be diffuse within the cytoplasm (Fig. 7b). mCh-TaMs1 signal was observed at the cell periphery and co-localized with the PIP_2_A-GFP plasma membrane marker [51] (Fig. 7c). This co-localization was confirmed in plasmolysed epidermal onion cells which allows the distinction between the plasma membrane and cell wall.

The requirement for each of the putative TaMs1 motifs (SP-LBD-GPI) for secretion and cell surface tethering was also demonstrated using truncated translation fusions transiently expressed in onion epidermal cells. We first tested the function of the N-terminal signal peptide (SP) using Ms1-SP. Ms1-SP fluorescence accumulated in the apoplast (Fig. 7d). This suggests that TaMs1 is targeted to the secretory pathway by the presence of the N-terminal signal peptide.

Finally, we studied the function of the pro-peptide GPI-anchor using Ms1∆LBD and Ms1∆GPI. Onion cells co-transformed with TaMs1 lacking the LBD and the plasma membrane intrinsic protein 2A (PIP_2_A-GFP) plasma membrane marker expressed fluorescence only at the outer surface of the plasma-membrane (Fig. 7e). Post plasmolysis treatment, RFP signal was detected both at the retracted cell membrane and on Hechtian strands which form a membrane-cell-wall continuum. In the absence of the pro-peptide GPI-anchor, Ms1∆LBD fluorescence was co-localized with the plasma membrane marker pre- and post-plasmolysis (Fig. 7f). Surprisingly, we additionally observed fluorescence within the cytosol. We interpret these findings to mean the GPI-anchor is required for specific targeting of TaMs1 to the plasma-membrane.

## Discussion

We previously reported the identification of *TaMs1*, a dominant wheat fertility gene sequence located on chromosome 4BS [16]. *TaMs1* was shown to be necessary for pollen exine formation. The phenotype for abnormal exine formation commonly leads to reduced fertility or complete male sterility. Pollen exine defective mutants have been reported to be a consequence of (i) defects in tapetal cell layer development, such as *tdr, tip2, eat1, ptc1* and *udt1* [14, 50, 52–54], (ii) disruption of the sporopollenin precursor synthesis and transport pathways, including *acos12, strl2, cyp703a3, psk1, dpw* and *abcg15* [5, 8, 13, 55–57] (iii) disruption of callose formation (*gls5)* [58], (iv) abnormal intine and premixine formation, such as *gt1, cap1 and dex1* [59–61], (v) and meiotic defects, including *xrcc3*, *zip4* and*pss1* [62–64]. These genes, whilst involved in different pathways, have demonstrated interdependent expression. For instance, rice *dpw* exhibits abnormal expression of *CYP704B2* [8], *np1* is misregulated in *TDR*, *DPW*, *CYP703A3, CYP704B2* and *ABCG15* expression [65], and loss-of-function mutants for *CYP703A3* were reported to have reduced expression of *DPW* and *CYP704B2* [66]. Furthermore, *TDR*, *EAT*, and *PTC1* had reduced expression in *tip2* [54], whereas abnormal expression of *CYP704B2, PTC1*, *PSK1,* and *DPW* was detected in *ptc1* anthers [54]*.* In order to determine whether *TaMs1* expression is dependent upon sporopollenin biosynthesis, we analyzed expression of wheat orthologues as well as rice sporopollenin biosynthetic genes in *ms1* anthers relative to WT. We determined that *TaMs1* was expressed earlier than sporopollenin precursor biosynthesis. However, to our surprise, the *ms1* mutation did not affect transcription levels of the biosynthetic genes during stages where they have previously reported to be essential for pollen development (Fig. 6; Additional file 3). Recently, *TaMs1* was shown to be up-regulated by heat-induced sterility in anthers containing uni-nucleate microspores compared with untreated anthers [67]. This suggests that whilst *TaMs1* is predominantly expressed during the early stages of pollen development (pre-meiosis and meiosis) under normal conditions, TaMs1 could play an important role post-meiosis, downstream of the biosynthetic genes listed genes in this study. Because *TaMs1’s* expression precedes that of genes involved in sporopollenin biosynthesis temporally, further experimentation is necessary to determine whether the TaMs1 protein itself persists past meiosis, the time of last detectable transcript expression, to coincide with the expression of these key sporopollenin biosynthetic genes. Importantly, the timing of expression of these wheat orthologues is in accordance with that reported in rice.

To date, wheat male sterile mutants linked to these rice genes have not yet been identified, with the exception of *TaCYP704B* [68]; this can in part be explained by genic redundancy embedded within wheat’s allohexaploid genome. However, given the advent of new genome-editing technologies with the capability of simultaneously generating loss-of-function mutants in a single transgenic event, there is the possibility of uncovering additional genes necessary for sporopollenin biosynthesis.

Recent evidence from Wang et al. (2017) suggests that TaMs1 (B genome) dominance in allohexaploid wheat is likely due to epigenetic repression of its homeoalleles [17]. Further, phytohormones play an essential role in the regulation of stamen and pollen development [69]. Here, we show the *TaMs1-B* promoter when compared to its homeologues contains several unique motifs with homology to ABA responsive (ABREOSRAB21), and jasmonate/ethylene responsive (GCCCORE-box) *cis*-elements (Fig. 2). We show that *TaMs1* expression is enhanced by ABA (Fig. 3), but not by JA exogenous treatment whereas treatments with hormones IAA and GA_3_ revealed *TaMs1* to be responsive only to auxin. Importantly, auxin has been reported as a key regulator at both early and latter stages of male gametogenesis, where it has been shown to be important for cellular differentiation, cell elongation and division [70]. ABA on the other hand, is suggested to act as a potential signal leading to male sterility [71]. Confirmation of the importance of such *cis*-elements in hormone response signaling during microsporogenesis requires further experimentation.

It is generally assumed that protein trafficking plays a central role for protein function. Here, we identified TaMs1 to contain two putative signal sequences: an N-terminal signal peptide (SP) and a C-terminal GPI-anchor pro-peptide (Fig. 7a). The SP is expected to target TaMs1 for translocation across the ER allowing the protein to enter the vesicular pathway [72], whereas the GPI anchor is expected to retain the mature protein at the extracellular side of the plasma membrane [73]. As expected, using transient expression of a TaMs1 translational fusion with mCherry in onion epidermal cells, we determined TaMs1 to be localized at the plasma membrane (Fig. 7c). In order to validate function of TaMs1’s predicted signal sequences for transport, we used truncated TaMs1 translational fusion proteins. The signal peptide alone was determined to induce protein secretion (Fig. 7d). Despite TaMs1 lacking the GPI-anchor pro-peptide, the truncated protein remained targeted to the plasma membrane, but was also detected to a lesser extent in the cytosol (Fig. 7f). This contrasts with AtLTP1 which, despite the absence of a GPI anchor, has only been identified at the plasma membrane [74]. Despite the fact that TaMs1’s GPI-anchor was not necessary for the protein to be targeted to the plasma membrane, it appears to be essential for its function. This is supported by the finding that *ms1j*, which contains a SNP converting Serine 195 to a Phenylalanine (S195F) is male sterile [see Additional file 4] [17]. Importantly, this residue is predicted to be at the omega cleavage site of the GPI-anchor pro-peptide and this point mutation results in the loss of potential C-terminal GPI-modification site [see Additional file 4]. Why TaMs1’s GPI-anchor pro-peptide is essential for the protein activity could be explained by the unique properties of GPI-anchors: (i) it has been proposed that the functional importance of the GPI anchor could be related to its characteristic to allow greater three-dimensional flexibility for the protein at the cellular surface [29]. (ii) Additionally, unlike transmembrane proteins, such GPI-anchored proteins have the potential to also be released from the cell surface via the activity of phospholipases [75]. Considering these properties, it is reasonable to assume that TaMs1 would be secreted from both the tapetal cell layer and developing microspores, and be tethered to the cell surface of each. Upon GPI-anchor cleavage by a phospholipase, TaMs1 could deliver sporopollenin precursors from the tapetal cell surface to the developing microspore surface. At this point, microspore derived TaMs1 proteins could potentially act as precursor receivers and therefore be responsible for the local deposition of exine at the cell surface. It is interesting that in a similar study, Wang et al.*,* reported TaMs1 to be localized to mitochondria in onion epidermal cells [17]. Relative to our findings of TaMs1 being localized at the cell surface, it is clear that further experimentation is necessary to determine where TaMs1 is localized *in planta*, particularly in wheat as opposed to interpretations based on an orthologous system like onion epidermal cells. Furthermore, validation of lipid binding by the TaMs1 fluorescent protein translational fusions is needed, as well as determining whether the translational fusions have the capacity to complement (i.e. restore male fertility) *ms1* mutants.

## Conclusions

In this study, we attempted to further understand the role of TaMs1 in relation to pollen exine formation. Our results provide new insight into the importance of GPI-anchored LTPs at the early stages of anther development. We also identified putative wheat orthologues of rice sporopollenin biosynthetic genes. Future studies on the functional role of TaMs1 in vivo are required to understand how this protein controls sporopollenin deposition onto the microspore in wheat.

## Additional files

Additional file 1: Primers used for qRT-PCR. (DOCX 15 kb)

Additional file 2: Callose deposition during meiosis in WT and *ms1d* mutant anther. Anthers containing meiocytes (mei), dyad, tetrad were dissected and stained by aniline blue solution. WT (top) and *ms1d* (bottom) samples for each stage were shown. Right panels show tetrad microspores undergoing callose wall degradation and transitioning to uninucleate microspore. Top is callose staining image and bottom is DIC image of same tetrad microspores. Bars in all panels = 50 μm. (DOCX 163 kb)

Additional file 3: List of selected genes reported to be required for male fertility in rice. (DOCX 20 kb)

Additional file 4: *ms1j* results in the loss of potential GPI-modification site. TaMs1 and Tams1j peptide sequences were tested for prediction of potential GPI-modification site using big-PI Plant Predictor (Eisenhaber et al., 2003). (DOCX 15 kb)
