# Supplementary material for: Wheat TaMs1 is a glycosylphosphatidylinositol-anchored lipid transfer protein necessary for pollen development
Source: BMC Plant Biol. 2018 Dec 5;18:332. doi: 10.1186/s12870-018-1557-1 (PMC6280385; doi:10.1186/s12870-018-1557-1)
Supplement: Supplementary file 1 — Primers used for qRT-PCR. (DOCX 15 kb) [file 12870_2018_1557_MOESM1_ESM.docx]

| **Gene** | **Forward primer (5’ to 3’)** | **Reverse primer (5’ to 3’)** |
| --- | --- | --- |
| *TaGAPdH* | TTCAACATCATTCCAAGCAGCA | CGGACAGCAAAACGACCAAG |
| *TaActin* | GACAATGGAACCGGAATGGTC | GTGTGATGCCAGATTTTCTCCAT |
| *Ta13-3-3* | ACGCAGCTACCTGTATCATTC | CGACGATGTCCACATGACC |
| *TaMs1_B* | CCTCTACATCATCCTCTGAGTCGC | GTACGAGCGGACAGAAACGATAG |
| *TaMs1_A* | CCTCTACATCATCCTCTGAGTCGC | TGAACATACTGCTGCTACCAGACACTA |
| *TaMs1_D* | CCTCTACATCATCCTCTGAGTGGC | TCCATACTCCTGCCAACGACAG |
| *TaABCG15* | CTCACCTACAACTGCGGGAG | AGAAGTAGGCGAGGAGGCG |
| *TaCYP703A3* | CGCCAGGCTCTTCCACTG | GCCTTGGGCATGGTCATC |
| *TaCYP704B2* | GTTCATCGACCCGCTGTG | ATGACGCTGTAGGTGAACTCG |
| *TaDPW* | CGCCAGCTACGTCGAGAC | CTGGTAGATGCTGCCGAGG |
| *TaPSK1* | AACACAGTCTTCTATGTGCTGGAG | CAAGATCAATCCCCACTCTTCC |

**Additional file 1: Primers used for qRT-PCR.**
