# Supplementary material for: Wheat TaMs1 is a glycosylphosphatidylinositol-anchored lipid transfer protein necessary for pollen development
Source: BMC Plant Biol. 2018 Dec 5;18:332. doi: 10.1186/s12870-018-1557-1 (PMC6280385; doi:10.1186/s12870-018-1557-1)
Supplement: Supplementary file 2 — Callose deposition during meiosis in WT and ms1d mutant anther. Anthers containing meiocytes (mei), dyad, tetrad were dissected and stained by aniline blue solution. WT (top) and ms1d (bottom) samples for each stage were shown. Right panels show tetrad microspores undergoing callose wall degradation and transitioning to uninucleate microspore. Top is callose staining image and bottom is DIC image of same tetrad microspores. Bars in all panels = 50 μm. (DOCX 163 kb) [file 12870_2018_1557_MOESM2_ESM.docx]

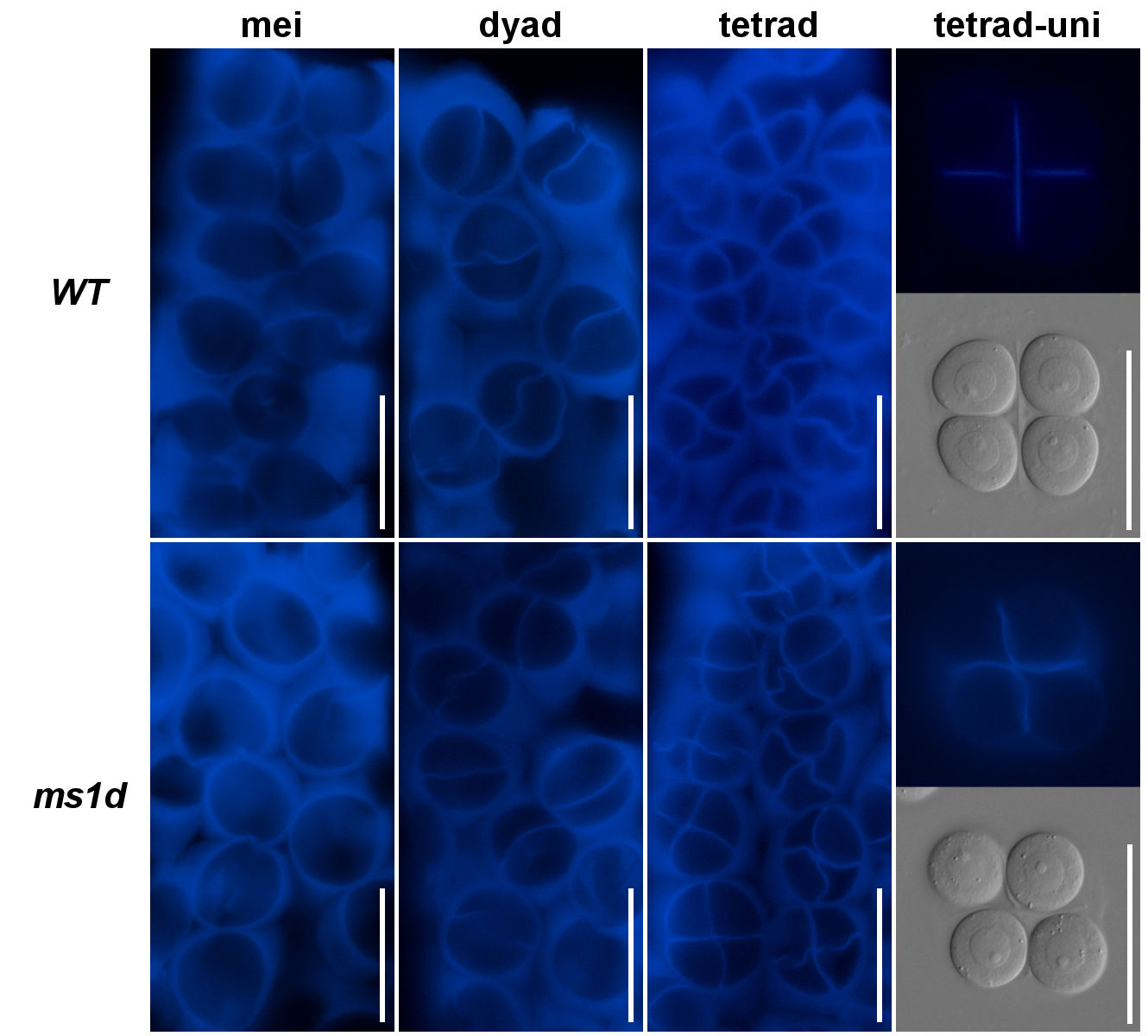


**Additional file 2: Callose deposition during meiosis in WT and *ms1d* mutant anther.** Anthers containing meiocytes (mei), dyad, tetrad were dissected and stained by aniline blue solution. WT (top) and *ms1d* (bottom) samples for each stage were shown. Right panels show tetrad microspores undergoing callose wall degradation and transitioning to uninucleate microspore. Top is callose staining image and bottom is DIC image of same tetrad microspores. Bars in all panels = 50 µm.
