# Supplementary material for: Wheat TaMs1 is a glycosylphosphatidylinositol-anchored lipid transfer protein necessary for pollen development
Source: BMC Plant Biol. 2018 Dec 5;18:332. doi: 10.1186/s12870-018-1557-1 (PMC6280385; doi:10.1186/s12870-018-1557-1)
Supplement: Supplementary file 3 — List of selected genes reported to be required for male fertility in rice. (DOCX 20 kb) [file 12870_2018_1557_MOESM3_ESM.docx]

**Additional file 3: List of selected genes reported to be required for male fertility in rice.**

| Pathway | Gene name | Protein encoded | Putative function based on rice orthologues | Reference |
| --- | --- | --- | --- | --- |
| Callose biosynthesis | *GLUCAN SYNTHASE-LIKE 5 (GSL5)* | Callose synthase | Essential for callose formation during microsporogenesis | Shi *et al*., (2015) |
| Primexine formation | *DEFECTIVE IN EXINE FORMATION1 (DEX1)* | Calcium-binding protein | Required for exine pattern formation | Yu et al., (2016) |
| Intine development | *GLYCOSYLTRANSFERASE1 (GT1)* | Glycosyl-transferases | Essential for intine formation and pollen development | Moon *et al*., (2013) |
|  | *COLLAPSED ABNORMAL POLLEN1 (CAP1)* | Arabinokinase-like protein | Involved in cell wall polysaccharides synthesis | Ueda et al., (2013) |
| Biosynthesis of sporopollenin | ACYL-COA SYNTHETASE 12 (*ACOS12*) | Fatty Acyl-CoA synthetase | Required for tapetum programmed cell death and male fertility. | Yang *et al*., (2017) |
|  | *STRICTOSIDINE SYNTHASE 2 (STRL2)* | Strictosidine synthase | Essential anther development and pollen wall formation | Zou *et al*., (2017) |
|  | NO POLLEN 1 (NP1) | Glucose-methanol-choline oxidoreductase | Essential for tapetum degeneration and pollen exine formation | Lui *et al.,* (2017) |
|  | CYTOCHROME P450 (*CYP703A3)* | Mid-chain fatty acid hydroxylase | Catalyzes in-chain hydroxylation of saturated medium-chain fatty acids | Aya *et al.,* (2009) |
|  | CYTOCHROME P450 (*CYP704B2)* | Fatty acid ω-hydroxylase | Catalyzes v-hydroxylation of long-chain fatty acids, implicating these molecules in sporopollenin synthesis | Li *et al*., (2010) |
|  | *POLYKETIDE SYNTHASE1 (PKS1)* | Polyketide synthase | Essential anther development and pollen wall formation | Wang *et al*., (2013) |
|  | *DEFECTIVE POLLEN WALL (DPW)* | Fatty acyl carrier protein reductase | Required for pollen exine development | Shi *et al.,* (2011) |
| Transporters | *ABC TRANSPORTER G FAMILY MEMBER 15 (ABCG15)* | ATP binding cassette transporter | Sporopollenin precursor transfer | Qin *et al.,* (2013) |
| Transcription factors | *TAPETUM DEGERATION RETARDATION (TDR)* | bHLH transcription factor | Master regulator necessary for pollen wall formation regulating tapetum development and degeneration | Zhang *et al*., (2008) |
|  | *UNDEVELOPED TAPETUM1 (UDT1)* | bHLH transcription factor | A crucial regulator of a genetic network that controls anther development and function | Jung *et al*., (2005) |
|  | *ETERNAL TAPETUM 1 (EAT1)* | bHLH transcription factor | Promotes aspartic proteases to trigger plant programmed cell death | Niu *et al*., (2012) |
|  | *TDR INTERACTING PROTEIN2 (TIP2)* | bHLH transcription factor | Promotes tapetal PCD and degeneration of callose surrounding the microspores | Ranjan *et al*., (2017) |
|  | *PERSISTENT TAPETAL CELL1 (PTC1)* | PHD finger protein | Implicated in pollen wall development by regulating tapetal PCD and breakdown. | Li *et al*., (2011) |
| Chromosome pairing and recombination | *X-RAY REPAIR CROSS COMPLEMENTING 3 (XRCC3)* | DNA repair protein | Essential for proper double-strand break repair and homologous recombination during meiosis | Zhang *et al*., (2015) |
|  | *ZRT-IRT-LIKE PROTEIN (ZIP4)* | Iron and zinc transporter protein | Promotes accurate synapsis and crossover of homologous chromosomes | Shen *et al*., (2012) |
|  | *POLLEN SEMI-STERILITY1 (PSS1)* | Kinesin-1-like protein | Essential for male meiotic chromosomal dynamics | Zhou *et al*., (2011) |
