# Supplementary material for: Wheat TaMs1 is a glycosylphosphatidylinositol-anchored lipid transfer protein necessary for pollen development
Source: BMC Plant Biol. 2018 Dec 5;18:332. doi: 10.1186/s12870-018-1557-1 (PMC6280385; doi:10.1186/s12870-018-1557-1)
Supplement: Supplementary file 4 — ms1j results in the loss of potential GPI-modification site. TaMs1 and Tams1j peptide sequences were tested for prediction of potential GPI-modification site using big-PI Plant Predictor (Eisenhaber et al., 2003). (DOCX 15 kb) [file 12870_2018_1557_MOESM4_ESM.docx]

| **Lines** | **Wild Type TaMs1** | ***ms1j* (S195F)** |
| --- | --- | --- |
| **Peptide sequence** | MERSRGLLLVAGLLAALLPAAAAQPGAPCEPALLATQVALFCAPDMPTAQCCEPVVAAVDLGGGVPCLCRVAAEPQLVMAGLNATHLLTLYSSCGGLRPGGAHLAAACEGPAPPAAVVSSPPPPPPPSAAPRRKQPAHDAPPPPPPSSEKPSSPPPSQDHDGAAPRAKAAPAQAATSTLAPAAAATAPPPQAPH**S**AAPTAPSKAAFFFVATAMLGLYIIL | MERSRGLLLVAGLLAALLPAAAAQPGAPCEPALLATQVALFCAPDMPTAQCCEPVVAAVDLGGGVPCLCRVAAEPQLVMAGLNATHLLTLYSSCGGLRPGGAHLAAACEGPAPPAAVVSSPPPPPPPSAAPRRKQPAHDAPPPPPPSSEKPSSPPPSQDHDGAAPRAKAAPAQAATSTLAPAAAATAPPPQAPHFAAPTAPSKAAFFFVATAMLGLYIIL |
| **Prediction of potential C-terminal GPi-modification site** | Potential GPI-modification site was found.  Quality of the site ............... : P  Sequence position of the omega-site :  **195**  Score of the best site ............ : 14.83 (P Value = 8.698e-07) | **None** potential GPI-modification site was found.  Among all positions checked, sequence position 197 had the best score. |

**Additional file 4: *ms1j* results in the loss of potential GPI-modification site.**

TaMs1 and Tams1j peptide sequences were tested for prediction of potential GPI-modification site using big-PI Plant Predictor (Eisenhaber *et al*., 2003).
